# Assessing the determinants of food choices among adolescents in India: A rainbow model using the socio-ecological framework

**DOI:** 10.1371/journal.pgph.0004776

**Published:** 2025-09-23

**Authors:** Kanishka Upadhyay, Bhavika Singhvi, Reshma Nakte, Mokalla Thirupathi Reddy, Hrusikesh Panda, Preetu Mishra, SubbaRao M. Gavaravarapu

**Affiliations:** 1 Nutrition Information, Communication and Health Education (NICHE) Division, ICMR - National Institute of Nutrition (ICMR-NIN), Hyderabad, Telangana, India; 2 Nutrition Specialist, United Nations Children’s Fund (UNICEF), India Country Office, New Delhi, Delhi, India; 3 Faculty of Medical Science, Academy of Scientific and Innovative Research (AcSIR), Ghaziabad, Uttar Pradesh, India; Universitat Rovira i Virgili, SPAIN

## Abstract

In India, the rising risk of overweight and obesity among adolescents is a significant public health concern, primarily associated with their frequent consumption of nutrient-poor snacks, sugar-sweetened beverages, and fast foods. Identifying the determinants of their food choices is crucial for developing effective nutrition promotion strategies. This study aimed to identify the determinants of food choices among adolescents in two metro cities in North and South India using a cross-sectional, mixed-methods approach. The study involved adolescents (n = 869) studying in 8^th^ and 9^th^ grades from randomly selected government and private schools, utilizing a pre-tested questionnaire and virtual food preference flip cards (quantitative phase), along with in-depth interviews with adolescents, teachers, and parents (n = 11) (qualitative phase). A four-level socio-ecological model was adopted to categorize the determinants associated with adolescent’s food choices. The findings revealed that taste (51%) was the primary determinant driving adolescents to choose outside foods, followed by peer influence (31%) and trendy foods (27%). In-depth interviews further highlighted the impact of social media, parental influence, celebrity endorsements, popular food trends, and marketing incentives such as toys and coupons on adolescents’ food choices. The study provides a springboard for future research into the development of a nutrition-friendly choice architecture to encourage adolescents to make healthy food choices. Public health policy should utilize these determinants to transform the existing food environment of adolescents.

## Introduction

Adolescence is a pivotal stage for the formation of eating habits, which often persist into adulthood. This period is critical as the food choices and behaviors established during adolescence can significantly impact long-term health outcomes [1]. According to a fact sheet released by the World Health Organization (WHO) in 2022, the global prevalence of overweight and obesity among children and adolescents aged 5–19 has risen sharply, increasing from 8% in 1990 to 20% in 2022 [2]. In India, this challenge is further intensified by the triple burden of malnutrition, which includes stunting, wasting, and micronutrient deficiencies, alongside a concerning rise in overweight and obesity among adolescents [3]. The Comprehensive National Nutrition Survey (CNNS) indicates that approximately 4% of Indian adolescents are overweight and obese. The risk of malnutrition and non-communicable diseases is high in this population due to dietary deficiencies and reliance on fried and junk foods. Therefore, it is crucial to focus on this age group, which comprises one-fifth of the country’s population, to achieve the demographic dividend [4] (p. 147).

There are various factors which influence the dietary intake and food choices of adolescents, including the specific food environment they are exposed to and from which they exercise food options [5]. Therefore, studying the food environment and determinants influencing adolescents is crucial to produce policy recommendations and design successful intervention programs [6].

The Socio-Ecological Model (SEM) for health promotion provides a useful framework for studying the determinants of food choices in adolescents at various levels and interactions within and outside their surroundings [7,8]. The SEM allows researchers to consider not only individual factors but also the broader social, environmental, and policy-related influences that shape behavior [9]. It has proven to be effective in supporting behavioral interventions, particularly those aimed at understanding and improving the food environment of adolescents [9–11]. This makes SEM particularly relevant to understand adolescent food choices, which are shaped by both internal preferences and external factors like peer pressure and digital food advertising [8,12].

The present study, through the lens of the SEM, aims to categorize the determinants of food choices into intrapersonal factors (taste, visual appeal, familiarity, attitudes, preference, and decision-making around food, and nutrition knowledge); interpersonal factors (parental interaction, school and peer influence); advertising and marketing strategies (trendy foods, celebrity endorsements, social media, branding, food product placement, and nutrition claims); and public policy (availability, affordability and accessibility) [10–15].

## Materials and methods

### Study design and population

This was a cross-sectional mixed-methods study consisting of a quantitative phase and a qualitative phase. The quantitative phase employed a questionnaire-based survey and virtual food preference flip cards (VFPFs). In the qualitative phase, semi-structured, in-depth interviews were conducted.

### Ethics statement

The study was approved by the Institutional Ethics Committee of the Indian Council of Medical Research – National Institute of Nutrition (ICMR-NIN), Hyderabad, India (approval no. 23/IVA/2022) on October 6, 2022. Written informed consent was obtained from school officials and students, and electronic consent from parents/guardians was facilitated through the class teacher for all participants.

### Study area

**Fig 1:**
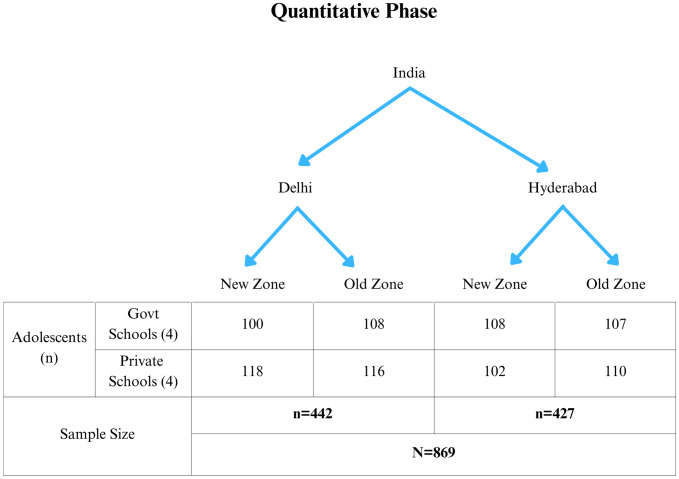
Sampling framework for the quantitative phase of the study.

The present study was conducted in two metro cities from North (Delhi) and South of India (Hyderabad). The cities were stratified into old city and new city zones. From each of the zones, a list of schools was prepared and approached as per convenience. From these, the first 20 schools that expressed interest in participating were shortlisted, including both government and private co-educational institutions. A random selection using the pick-of-lots method was then conducted to choose one government and one private school per zone, as they cater to different socio-economic groups [16]. Schools that agreed to participate were included in the study after obtaining the necessary institutional permissions and informed consent. A total of four schools were selected from each city, compiling to n = 8 schools in total (Fig 1).

School officials and students provided written informed consent, and the class teacher obtained electronic consent from parents to sign as guardians for all students. They were able to withdraw from the study at any time without giving a reason.

### Recruitment of the participants

The quantitative phase of the study included adolescents studying in 8th and 9th grade (N = 869). The participants were between 11 and 16 years of age. Semi-structured, qualitative interviews were conducted with adolescents, teachers, and parents in Hyderabad. Participants were selected from randomly selected schools based on their availability and willingness to participate. The participants included 5 adolescents (3 from government schools and 2 from private schools), 3 teachers (two from government school, and 1 from private school), and 3 parents.

### Research tools

#### Questionnaire.

In the quantitative phase of the study, a pre-coded, close-ended pen-and-paper questionnaire (27 items) was developed after conducting an extensive review of literature on adolescent food choices [8–30]. The questionnaire was developed in English using SEM. The general characteristics of the participants were recorded, followed by assessing the determinants of food choices at four levels—intrapersonal, interpersonal, advertising and marketing strategies, and public policy factors (Table 1). Constructs under each domain included taste and visual appeal (intrapersonal), peer influence (interpersonal), exposure to advertisements, celebrity endorsements, trendy foods, marketing offers, ‘Scratch-and-Win’ promotional offers (advertising and marketing), and food affordability and availability (public policy). The responses were recorded on a 4-point Likert scale with options: “Always,” “Sometimes,” “Rarely,” and “Never,”.

**Table 1:** Development of research tools using SEM framework.

SEM Domain	Component in the Questionnaire	Component in the Virtual Food Preference Flip cards (VFPFs)	Themes in In-Depth Interviews
**Intrapersonal Factors**	Taste, Visual appeal	Convenience (Picture A), Visual appeal (Picture B)	Taste, Visual appeal, Familiarity, Convenience
**Interpersonal Factors**	Peer influence	Not assessed	Peer influence, Parental influence, Teacher/school influence
**Advertising & Marketing**	Trendy foods, Brand names, Advertisements, Celebrity endorsements, Promotional offers (Buy1Get1Free, Scratch-and-win)	Nutrition claims (Picture C), Buy1Get1Free offer (Picture D), Food product placement (Picture E)	Social media influence, Brand appeal, Advertisements, Celebrity endorsements, Promotional offers, Trend-driven food choices
**Public Policy Factors**	Affordability, Availability	Not assessed	Availability, Affordability, Accessibility,

A panel of five experts from diverse fields- nutritionists (n = 2), a physician, a psychologist, and a social scientist, evaluated the contents of the questionnaire for their relevance, clarity, simplicity, and ambiguity. Subsequently, the questionnaire was pre-tested among 10 adolescents each in Delhi and Hyderabad. Based on the pre-test results, modifications were carried out in the questionnaire along with the use of simple language to make it easy to comprehend.

#### Virtual food preference flip cards (VFPFs).

VFPF’s were developed to simulate real-life food choice scenarios and help participants actively engage in decision-making. These flip cards were designed to trigger memory recall and enable adolescents to express preferences in a setting that reflected typical food environments.

Following a thorough review of literature [15,20–25], five VFPFs were created by the researcher using MS Paint (Microsoft Paint, a basic graphics editor) and reviewed by three experts in the field of nutrition. Each flip card consisted of a pair of contrasting food pictures, with a total of twelve colorful graphics representing specific determinants of food choice, mapped to the appropriate SEM domains (Table 1).

Picture A highlighted convenience as a determinant of food choice by showing a whole mango, mango juice, mango jelly, and sliced mango pieces. To examine visual appeal as a determinant, picture B presented a chapati (flatbread) and a shawarma (flatbread filled with vegetables and meat) and/or vegetable roll. Picture C depicted nutrition claims such as “multigrain” and “non-fried.” Picture D displayed two packets of chips, one without the “Buy 1 Get 1 Free” offer and the other with the offer. Lastly, picture E explored food product placement, with fruits placed in front of less healthy burgers in one food stall and behind the burgers in another.

#### In-depth interviews.

The theme guide for in-depth interviews was developed based on a review of literature [13,17–19]. A deductive coding framework [20] was used to explore predefined themes that aligned with the SEM framework (Table 1) to ensure comprehensive understanding of food choices among adolescents. The theme guide was pre-tested with individuals from each respondent group, and necessary feedback was incorporated to refine a few questions.

### Data collection and analysis

#### Survey questionnaire and VFPFs.

The objective of the study was explained to the participants through a classroom lecture. The investigator verbally dictated each question in English and clarified doubts, if any. The questionnaire was self-administered and completed by the participants in their respective classrooms under the supervision of the investigator. Following this, the VFPFs were distributed to each of the participants. They were instructed to select their preferred food items and mark “✓” in the boxes given below.

The quantitative data was coded, entered into Microsoft Excel, and analysed using SPSS (Version 23.0. SPSS Inc., Chicago, IL, USA). Descriptive statistics (percentages, standard deviation and mean) were used to assess frequency. The Chi-square test (non-parametric test) was used to study the relationship between variables under study.

#### Conducting in-depth interviews.

The participants were informed about the study objectives via telephonic conversation. Trained professionals, including a moderator and a note-taker proficient in Telugu (the local language), Hindi, or English, conducted the interviews face-to-face. With the permission of the participants, the interviews were recorded using an audio recorder on a mobile phone to ensure detailed documentation of all provided information for subsequent analysis. The interviewer ensured that the respondents felt relaxed and comfortable to make the conversation easier and to freely express his/her thoughts. After an informal introduction, basic questions were asked, leading to more comprehensive questions to obtain in-depth information.

#### Thematic analysis.

The qualitative data were analysed using Braun and Clarke’s six-phase framework for thematic analysis [20]. The audio-recorded interviews were transcribed verbatim and anonymised. Two independent researchers familiarised themselves with the transcripts through repeated reading and then manually coded the data using the pre-established thematic framework. The coding process was iterative and interpretative, with careful attention paid to preserving the meaning and context of participant responses. Transcripts were then compiled into individual analytical reports. Within each report, raw data were classified into conceptual levels. Representative quotes were extracted to illustrate key findings, and contradictory perspectives were also flagged to ensure diversity of views and analytical balance. Although inter-coder reliability was not statistically calculated, analytic rigor was ensured through a systematic process of collaborative review, comparison, and resolution of coding discrepancies between the two coders.

## Results

A total of 869 adolescents were surveyed, with 443 (51%) females and 426 (49%) males aged between 11–16 years. Generally, this age group corresponds to standard 8^th^ and 9^th^ in government schools in the Indian education system [16].

The results have been presented through the SEM perspective: Intrapersonal, Interpersonal, Advertising and marketing, and Public policy.

### Intrapersonal determinants

**Fig 2:**
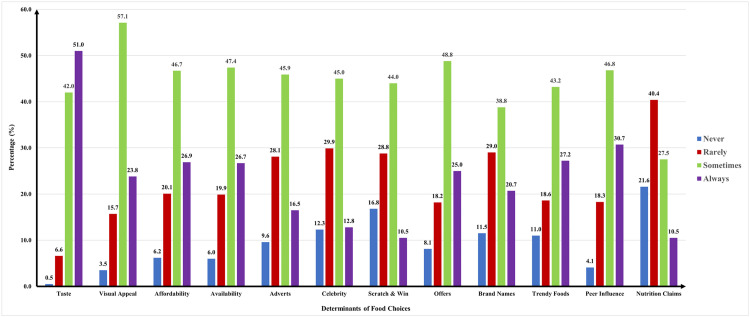
The determinants of food choices among adolescents (n = 869) in both the cities (pooled data).

The pooled data of both the cities revealed that taste (51%) was the primary determinant for adolescents to purchase outside foods. Other determinants like visual appeal was 24% in the current population (Fig 2).

**Fig 3:**
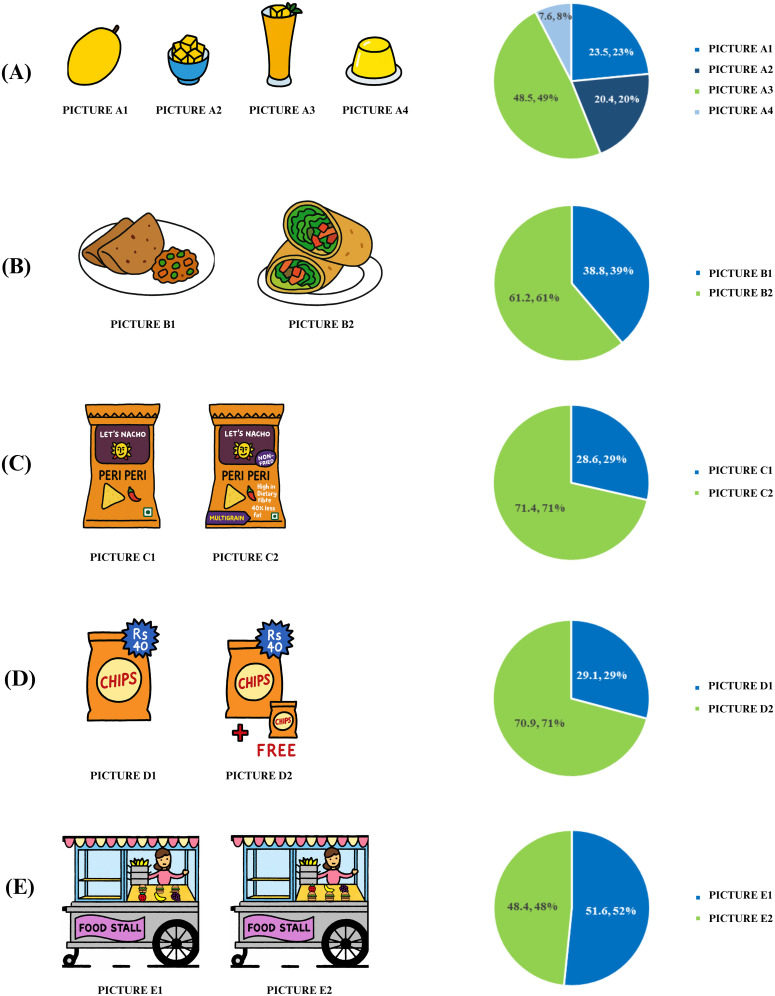
Virtual food preference flip cards depicting food choice determinants. Description: Convenience- a whole mango (A1), mango juice (A2), mango jelly (A3), and sliced mango pieces (A4); Visual appeal- a chapati (flatbread, B1) and a shawarma roll (flatbread filled with meat and vegetables, B2) and/or vegetable roll; Nutrition claims- C1with no nutrition claim, C2 with nutrition claims such as “Multigrain” and “Non-fried”; Offers- D1 with no offer, D2 with Buy 1 Get1Free offer; Food product placement- Fruits placed in front of burgers (Picture E1), fruits placed behind the burgers (Picture E2). Please note the corresponding pie charts are displayed alongside the flip cards. * All panels are original illustrations created by the author (K.U.) using Microsoft Paint.

The VFPF’s presented to the adolescents from both schools, indicated that 71% of them opted for a healthier choice (picture C2) when nutrition claims such as “multigrain” and “high in dietary fibre” were highlighted. Additionally, an equivalent percentage of adolescents were swayed by Buy1Get1Free offers (picture D2). About 52% of adolescents responded favourably to picture E1, where healthy foods (fruits) were placed in front of less healthy options (burgers) (Fig 3).

### Interpersonal determinants

The pooled data of both cities revealed that the second major determinant was peer influence (30.7%) in the current population (Fig 2).

### Advertising and marketing strategies

In the pooled data from both cities, trendy foods emerged as the third significant determinant (27.2%), followed by promotional offers such as buy-one-get-one-free (25%), brand names (20.7%), advertisements (16.5%), celebrity endorsements (12.8%), and scratch-and-win promotions (10.5%) (Fig 2).

### Public policy

Affordability (26.9%) and availability (26.7%) were also important determinants of food choices among adolescents in both the cities (Fig 2).

### In-depth interviews

In-depth interviews were conducted with adolescents, teachers, and parents, exploring themes related to factors affecting adolescent food choices (Tables 2–4).

**Table 2:** Themes and key findings from in-depth interviews with adolescents.

Themes	Key Findings	Representative Quotes
Major prompts while selecting foods	Adolescents often prioritise taste and affordability when selecting packaged foods. Decisions are made based on sensory appeal and value for money, particularly among school-going adolescents with limited pocket money.	*“Taste is the first thing I look for. Also, while buying a pack of Kurkure or Fun Flips, I check how much quantity of chips is inside. If it’s pricey, I won’t buy it. If it’s cheap, I’ll buy it.”* ***-12-year-old girl*** *“My parents give me limited money, so I always go for the snacks that taste good and are not overpriced.”* ***– 13-year-old boy***
Peer Influence	Adolescents often follow peer-led decisions when eating out, with one group member typically choosing what to order. Their choices are influenced by price, visual appeal, and popular food trends, often discovered through food bloggers or social media.	*“We look at the menu first and see the pictures of dishes. If we can afford it, then we’ll buy it. We do try new dishes sometimes, mostly we like trying Chinese food and chaats.”****-14-year-old girl*** *“There’s one guy in our group who always decides what we order. If he sees something trending or new on YouTube, we all try it together.”* ***– 15-year-old boy***
Food Advertisement and Marketing	Adolescents often buy food products endorsed by their favourite celebrities, with marketing tactics using emotional appeals like fun and fantasy. Promotion of High in Fat, Sugar, and Salt (HFSS) foods involves offering freebies and discounts.	*“I remember Virat Kohli’s ‘Too Yumm’ advertisement. I don’t always buy it, but I like the different flavours, especially the latest dahi-papdi flavour. Last time, I got a free scratch card and I was happy.”-* ***14-year-old girl***
Social media	Adolescents regularly watch trendy food cooking videos on YouTube and follow food bloggers. They engage in virtual communities to share their experiences with new dishes.	*“I like watching ‘MasterChef India’ and food bloggers in Hyderabad. They keep on posting new places to eat and what all to try. So, friends and I decide on a place to eat on that basis. Sometimes, if I want my mother to try making a new dish, then I show her cooking videos.”-* ***14-year-old girl***

**Table 3:** Themes and key findings from in-depth interviews with teachers.

Themes	Key Findings	Representative Quotes
Food habits and choices of today’s adolescents	Adolescents opt for junk foods due to media influence, prioritize taste, and choose “cool” and “trendy” items to fit in their peer group.	*“I believe it’s important to tell parents not to pack such foods in tiffin carriers (lunch box), even if their child asks for them. We actually address this during PTM. Just yesterday, there’s this lady who works here, and her boy is in the 8th grade. He often brings jellies and chocolates to school. I strictly told his mother yesterday not to send such items to school.”* ***-56-year-old, Science teacher***
Foods sold by school canteens/street food vendors outside schools	Healthy foods are costlier than HFSS options, calling for school canteen menu adjustments to swap juices and soft drinks with fruits or custard. Government school mid-day meals include eggs, bananas, rice, and dal.	*“Our school canteen sells samosas, mysore bonda, cold drinks and all such junk food items. We’ve told the cook to sell items like Veg sandwiches, corn chaat etc. Also, few students get money to buy from canteen frequently. I tell them once a month its’ okay, but not regularly.”* ***-62-year-old, School Principal***
Role of teachers in inculcating healthy food habits	Teachers play a crucial role by monitoring students’ lunchboxes, organizing nutrition games and camps, appointing nutrition leaders to limit junk food consumption, and enforcing rules like providing a daily fruit.	*“Last year, students from National Institute of Nutrition came and explained about Anemia, and also conducted quiz. Many 9*^*th*^ *grade students participated, showing interest and gaining knowledge.”****-47-year-old, Language Teacher***
Nutrition component in school curriculum	To integrate nutrition effectively into the school curriculum, it should begin in nursery classes through creative means like poems and fun games, as currently, teachers only address it within specific chapters due to time constraints. Practical application of theoretical knowledge is also essential.	*“It’s all bookish information that’s given. It’s important to follow, but since some parents are illiterate, they should first be made aware of healthy and unhealthy foods. I also feel that primary school children are forgetful and won’t remember which food provides what, so it’s important to teach them practically, maybe through poems, fun activities, and similar methods.” -* ***62-year-old, School Principal***

**Table 4:** Themes and key findings from in-depth interviews with parents.

Themes	Key findings	Representative quotes
Role of parents in shaping healthy food habits among kids	Working mothers often opt for packaged foods or provide money for outside meals due to time constraints. Implementing home food rules can guide adolescents in making mindful dietary decisions.	*“My kids like chaat items like pani-puri and refined foods like cakes, bread, pizza, and burgers. I have to go to the office at 7 am, and sometimes it’s difficult to cook, so I keep bread, jam, and namkeen to ensure they at least eat something.”* ***-42-year-old Mother***
Family interaction around food	Family meals promote better food choices among adolescents, as parental engagement around food influences their decisions.	*“Children learn food habits from their family only. They see what type of food their mother, father, elder sister/brother are eating and how they are eating.”* ***-46-year-old Father***
Major prompts for adolescents	Adolescents are influenced by TV and newspaper advertisements, trendy food items, convenience, visual appeal, and marketing incentives like toys and coupons, all while seeking food that satisfies their taste preferences.	*“I think when children look at ads on TV and newspapers and on hoardings these days, they get curious. I remember my son once told me that he wants to try Momo Burger (laughs) just because he saw an ad in the newspaper, even though the name sounded strange to me.”* *-* ***38-year-old Mother***
Impact of screen time on eating pattern of adolescents	Adolescents tend to overeat when eating in front of TV or mobile screens.	*“While watching TV, they don’t even notice what they are eating. If I give my son beans curry, which he dislikes, he will eat it without complaining if he’s watching his favorite show! Sometimes, he forgets to chew properly, drinks lots of water in between, and then says, ‘Mummy, I’m done.’”* *-* ***42-year-old Mother***

Key findings indicate that adolescents make food choices majorly based on determinants such as taste, visual appeal, familiarity and preference for food. Apart from these, advertising and marketing also have an impact on their food choices. Brand names, nutrition claims, celebrity endorsements with emotional appeal, marketing strategies for HFSS foods, and offers such as freebies and discounts further influence their purchasing decisions. Adolescents followed food trends on YouTube and blogs, participated in virtual communities to share experiences with new dishes. One adolescent reported, *“I watch ‘Master Chef India’ and follow food bloggers in Hyderabad to find new places to eat. I also show my mom cooking videos when I want her to try a new dish.”*

Teachers highlighted that adolescents tend to eat junk food due to its taste and also to become “cool” and “trendy” in their peer circle. Foods sold in school canteens are usually HFSS which are costly as compared to healthy food options. One teacher mentioned *“Our school canteen offers items like samosas, mysore bonda and cold drinks. We’ve asked the cook to provide healthier options like Veg sandwiches and corn chaat, and I advise students to buy from the canteen only occasionally.”* The school principal emphasised that teachers can play a pivotal role by supervising lunch choices, organising nutrition-focused activities using creative methods like jingles, poems or games, appointing nutrition leaders, and offering daily fruit breaks.

According to parents, adolescents are prompted by visual appeal of foods, TV and newspaper advertisements, and marketing incentives like toys and coupons. One parent remarked *“I think when children look at ads on television, newspapers and on hoardings these days, they get curious. I remember my son once told me he wants to try Momo Burger (laughs) just because he saw an ad in the newspaper, even though the name sounded strange to me.”* Due to time constraints, working mothers frequently choose packaged foods or offer money for meals outside the home.

A significant relationship was found between mothers’ educational status and food product price (χ² = 36.44, p = 0.002) and father’s educational status and food availability (χ² = 36.75, p = 0.001) (Table 5).

**Table 5:** Chi-square analysis of the relationship between parental educational satus (n = 869) and food price and availability.

Parental Education Level	Response	Food product price (Mother) n (%)	Food Availability (Father) n (%)
No formal education	Always	23 (32.9%)	17 (47.2%)
	Sometimes	33 (47.1%)	12 (33.3%)
	Rarely	10 (14.3%)	4 (11.1%)
	Never	4 (5.7%)	3 (8.3%)
Primary school	Always	39 (31.2%)	26 (32.5%)
	Sometimes	49 (39.2%)	27 (33.8%)
	Rarely	24 (19.2%)	20 (25.0%)
	Never	13 (10.4%)	7 (8.8%)
Secondary school	Always	29 (21.0%)	33 (20.2%)
	Sometimes	76 (55.1%)	81 (49.7%)
	Rarely	25 (18.1%)	41 (25.2%)
	Never	8 (5.8%)	8 (4.9%)
High school	Always	79 (30.6%)	70 (25.8%)
	Sometimes	126 (48.8%)	133 (49.1%)
	Rarely	35 (13.6%)	45 (16.6%)
	Never	18 (7.0%)	23 (8.5%)
Vocational training	Always	5 (26.3%)	0 (0.0%)
	Sometimes	8 (42.1%)	11 (64.7%)
	Rarely	4 (21.1%)	4 (23.5%)
	Never	2 (10.5%)	2 (11.8%)
Graduate/Postgraduate	Always	59 (22.9%)	86 (28.5%)
	Sometimes	114 (44.2%)	148 (49.0%)
	Rarely	76 (29.5%)	59 (19.5%)
	Never	9 (3.5%)	9 (3.0%)
χ² (20)		χ² = 36.44, p = 0.002	χ² = 36.75, p = 0.001

* Frequencies are presented with row percentages in parentheses. Chi-square tests indicate statistically significant associations between mothers’ education and perceived food price concern and, between fathers’ education and reported food availability.

## Discussion

The present study explored the determinants of food choices among Indian adolescents using a socio-ecological framework. The findings from the quantitative data of our study showed that taste alongside peer influence, and perceived trendiness of foods are the key determinants of food choices outside home among adolescents.

### Intrapersonal determinants

Taste emerged as the dominant proximal driver of food choice in both the quantitative and qualitative data. These findings are consistent with existing Indian evidence that highlights the strong role of sensory appeal and hedonic factors in adolescent food consumption patterns. A cross-sectional survey conducted in Karnataka among adolescents (n = 399; 13–16 years) reported a high intake of energy-dense processed and ready-to-eat foods, with preferences largely shaped by the appeal of palatability and convenience [21]. Similarly, an analysis of school canteen purchases in Mumbai (n = 300; 10–12 years) revealed that HFSS food dominated overall sales, with taste emerging as the key determinant of food choice [11].

Convenience also emerged as an important determinant. A study done in the United States investigated the selection of oranges (whole, fruit, sliced, or orange juice) by high school students during lunchtime in a cafeteria. The results indicated a notable preference for sliced oranges, with 16.2% of adolescents selecting them compared to 5.5% who chose whole oranges. This indicates strong preference for the convenience and accessibility of sliced fruit [23]. In our study, mango was presented in four different forms: whole fruit, sliced, mango juice, and jelly. We found that 49% of adolescents favoured mango juice over whole fruit (23%) as it’s more convenient.

Visual appeal was also shown to influence adolescents’ food choices. A recent Indian study demonstrated that digital promotion of attractive food images increased adolescents’ cravings and intent to consume both healthy and unhealthy foods, depending on the sensory cues highlighted [24]. This finding supports our result that adolescents were more inclined to select visually appealing fast foods, such as rolls and shawarmas, over traditional staples like chapati and curry. This suggests that presenting healthier food options in an enticing or visually appealing way holds the potential to influence adolescents to make healthy dietary choices [23]. Such positive sensory cues increase’s adolescent attention and emotions towards pro-nutritional images driving them towards healthy food selection [24].

### Interpersonal determinants

Interpersonal influences, particularly those exerted by peers, also emerged as a prominent driver of food choice. Our study’s findings are consistent with limited research conducted earlier in India which indicated that adolescents consume HFSS foods to conform to peer group norms [18]. Previous research from urban Indian schools similarly reported that adolescents frequently consume fast foods to align with group norms and to avoid social exclusion, even when they personally preferred homemade foods [4]. In-depth interviews in our study further underscore the influence of the desire to ‘fit in’ with peers and the aspiration to become ‘peer leaders’ in food choices made along with friends. Adolescents employ food choices as a means of crafting a desired image, forming judgments about others, and gaining acceptance within their peer groups [18,26].

Parental influence and household environment are also critical determinants. In-depth interviews in our study highlighted the challenges faced by working parents in preparing nutritious meals for their children, leading them to rely on instant and packaged foods. Similar findings were reported in an earlier study, where working parents find it difficult to prepare fresh meals as it demands more time and effort, involving tasks such as washing, chopping, and cooking, leading to reliance on ready-to-eat options [28]. A study conducted in Poland revealed that parents’ hectic schedules and long working hours hinder their ability to prepare homemade family meals [26]. Consequently, adolescents are inclined to consume fast and packaged foods rather than fresh alternatives.

Furthermore, the study found a significant association between mothers’ education and adolescents’ perception of food price as a concern. Earlier studies have documented the influential role of maternal education in shaping household food purchasing behavior. Educated mothers are generally more aware of nutritional information, pricing strategies, and the long-term health implications of food choices [14,17].

### Advertising and marketing determinants

The influence of advertising, particularly through television and social media, is also noteworthy. Our study revealed that adolescents were strongly influenced by promotional offers, celebrity endorsements, and food trends on social media. A study conducted in Pune reported that advertising strategies, particularly endorsements by film actors and sports celebrities, substantially shaped adolescents’ food preferences and brand loyalty [29]. Our in-depth interviews also revealed that many adolescents referred to YouTube food bloggers and popular cookery shows. This illustrates that media-mediated exposures often can translate into real-world consumption choices. Additionally, our quantitative data emphasises that a notable number of adolescents encounter food advertisements on social media, which subsequently leads to increased purchases of snacks and ice cream.

In an Australian study focusing on the impact of trendy food presentation, it was observed that when food items were labelled with attention-grabbing names, associated with celebrities, or promoted by influencers and advertisements, a significant proportion of adolescents were inclined to select these items [29]. Our research yielded congruent outcomes, with adolescents choosing specific food items due to their trendiness and social pressure to explore the latest culinary offerings. Furthermore, insights from our in-depth interviews highlighted that the mere mention of trendy food names, such as “Momo Burger”, elicited curiosity prompting adolescents to experiment with these HFSS foods, thereby contributing to their consumption.

Numerous studies underscore the significant impact of social media food content on adolescents’ dietary choices [29–31]. It was found that children aged 10–16 who watched food brand videos on YouTube or saw their favourite food brands advertised online were more likely to frequently consume unhealthy foods and drinks [32]. Our qualitative findings support this, showing that adolescents heavily rely on social media and food bloggers for recommendations on new and trendy cuisines. They frequently engage with cooking videos featuring popular food and cuisines, with YouTube being their preferred platform. Additionally, many adolescents actively participate in virtual communities, where they share and discuss their favourite culinary discoveries.

Food product placement strategies can effectively highlight healthier food options by positioning them prominently, such as at the front of the line, ahead of other choices at kiosks, or near checkout counters [22,33,34]. A study in Germany observed that relocating chips to a more distant location resulted in a decline in chip selection and an increased preference for starch-rich foods that remained within proximity [35]. Our study findings align with this research, demonstrating a similar trend among adolescents. Specifically, the majority of adolescents exhibited a preference for the target food (fruits) in the flip card when it was strategically placed in front of less healthy options like burgers. This suggests that priming healthy foods in settings like bakeries, school canteens, and other locations can potentially stimulate greater demand for nutritious choices over less healthy alternatives [23,36].

### Public policy determinants

The affordability of more expensive, healthier food options is a significant concern for adolescents. Convenience and the price of healthy foods often act as barriers to making healthy food choices [1]. A cross-sectional study among adolescents in Nepal revealed high consumption of junk foods in both public and private schools due to their easy availability and accessibility in school canteens [37]. Similarly, our interviews with teachers highlight that in the school environment, healthy foods are more expensive than HFSS foods in the canteen, and street food outside the school is also readily accessible.

On comparing qualitative data with quantitative data, it reflects that the motivation underlying making any food choice alters between four different realms of the SEM model. In-depth interviews facilitated identifying and understanding adolescents’ perception and their surrounding food environment through various stakeholders, which was not as definite as quantitative data. Altering a determinant at any given level can influence the food choices of adolescents. Hence, the unavoidable interplay between these factors should not be undermined while planning adolescent intervention programs.

**Fig 4:**
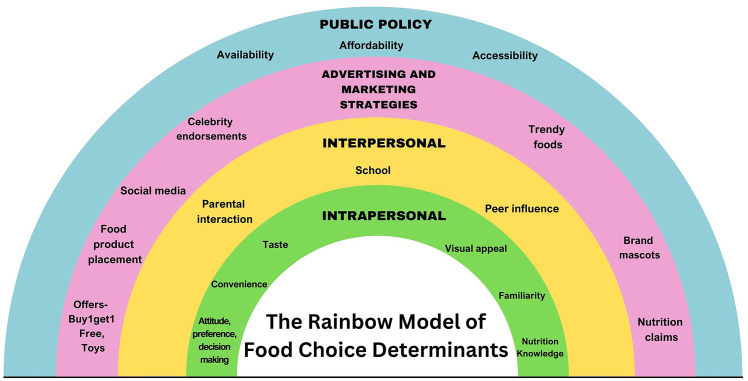
The rainbow model of food choice determinants. Description: This model depicts 4 dimensions-namely intrapersonal, interpersonal, advertising and marketing strategies, and public policy, and their interplay in influencing food choices in adolescents.

Based on these findings, the researchers propose the ‘Rainbow Model of Food Choice Determinants’*,* which offers a comprehensive framework encompassing multiple levels of influence—namely intrapersonal, interpersonal, advertising and marketing strategies, and public policy. This model illustrates the complex interplay of factors that shape the food choices of adolescents (Fig 4). While other established frameworks, such as Contento’s model [38], conceptualize food choice behavior across multiple ecological domains (cognitive, emotional, and behavioral factors), the Rainbow model has been specifically developed to reflect the Indian adolescent food choice scenario, offering a more contextually appropriate and practical framework. The Rainbow model provides a holistic understanding of the determinants of food choices among Indian urban adolescents, aligning with the socio-ecological framework used in this study.

This study has certain limitations. It was conducted only in two metropolitan cities, which may limit the generalisability of findings to adolescents in rural or peri-urban settings where food environments differ. Although the cities were stratified by region and both government as well as private schools were included to capture socio-economic variation, the limited number of school clusters precluded robust statistical comparisons between school types. Broadly similar patterns of determinants were observed across the groups, with taste emerging as the predominant factor followed by peer influence and trendiness of foods, yet formal testing could not be undertaken. In addition, reliance on self-reported data may have introduced recall and social desirability bias. Despite these limitations, the mixed-methods design and use of a socio-ecological framework provide a comprehensive understanding of adolescent food choice determinants in the urban Indian context.

In the context of existing scattered studies in India on the key determinants of food choice, our research provides valuable insights and lays the groundwork for comprehensive programs. Although this study represents an initial attempt to systematically examine the determinants of food choices among adolescents, it offers a framework that is likely applicable across major urban centres in the country. Understanding these determinants is crucial for gaining a new perspective on the food environment. Furthermore, similar food options are increasingly available in rural areas, with the year-on-year growth of processed foods. Therefore, it is imperative to investigate the factors influencing food choices among adolescents in these rural regions as well.

The findings of this study carry important implications for public health policy and school-based nutrition programs. Policymakers should consider integrating nutrition literacy into school curriculum, regulating food marketing targeting adolescents, and ensuring that school canteens promote healthy, affordable food choices.

## Conclusion and implications

This study provides an in-depth understanding of the determinants influencing food choices among Indian adolescents. Findings from both quantitative and qualitative phases consistently highlighted that taste, peer influence, and the appeal of trendy foods are primary drivers of adolescent food choices. Additionally, the influence of social media, celebrity endorsements, promotional offers, and marketing strategies for HFSS foods emerged as significant contributors. Public policy factors such as availability and affordability also shaped adolescent decisions.

These insights underscore the complex and interconnected determinants of adolescent eating behavior, as captured in the proposed Rainbow Model. This model, grounded in the socio-ecological framework, offers a practical and context-specific approach, and can be used to inform the design of targeted nutrition interventions in Indian settings by clearly mapping the key determinants of food choices.

Given the widespread exposure of adolescents to digital marketing and unhealthy food environments, particularly in school settings, there is an urgent need for comprehensive programs that address these determinants at all levels. Involving teachers and parents in promoting food literacy, regulating marketing practices, and improving school food environments is crucial. The findings also align with UNICEF policy recommendations to create healthier food systems for children and adolescents [39,40].

The study’s results can be beneficial in understanding the new perspective and expanding dimensions of the food environment. Given the growing availability and consumption of processed foods in rural India, this study can further be scaled up in these regions. The findings from this research will aid in developing a supportive, nutrition-choice architecture and nudge-based intervention programs, fostering healthier dietary choices among adolescents.

## Supporting information

S1 Data Comprehensive dataset and study instruments. This Excel file contains the full de-identified dataset and supporting documentation for the study, organized into multiple sheets as follows: **Sheet 1: Pooled Coded Data**. Contains the complete coded and de-identified dataset used for primary analyses, including participant demographics, survey responses, and all initial coded variables. **Sheet 2: Determinants Pooled Data**. Presents the pooled data (n = 869) on the determinants of food choices among adolescents from both study cities. This sheet includes the specific data points and summary statistics that correspond to the determinants of food choices, providing the underlying data for related analyses and visualizations. **Sheet 3: Codes.** Provides a comprehensive list of all codes and coding schemes applied to the dataset, including definitions and categories used for data analysis and interpretation. **Sheet 4: Chi-Square Test Results**. Presents the detailed results of Chi-square analyses, specifically examining the relationship between parental educational status (n = 869) and food price and availability. **Sheet 4: VFPFs Data**. Contains the raw response data from the Virtual Food Preference Flip Cards (VFPFs), detailing participant choices and preferences, and includes an embedded pie chart visualization generated from this data. **Sheet 5: In-depth Interview Questions**. Lists the complete set of guiding questions used during the in-depth interviews with study participants, serving as the detailed instrument for qualitative data collection.
(XLSX)
